# A cross‐sectional analysis of TikTok's most popular dermal filler videos

**DOI:** 10.1002/ski2.390

**Published:** 2024-05-06

**Authors:** Heloise de Baun, Patricia Cerri‐Droz, Samavia Khan, David Alper, Babar Rao

**Affiliations:** ^1^ Rao Dermatology Atlantic Highlands New Jersey USA; ^2^ Renaissance School of Medicine at Stony Brook University Stony Brook New York USA; ^3^ Center for Dermatology Rutgers Robert Wood Johnson Medical School Somerset New Jersey USA; ^4^ New York Medical College School of Medicine Valhalla New York USA; ^5^ Department of Dermatology Weill Cornell Medicine New York New York USA

## Abstract

**Background:**

Dermal filler injections pose serious risks when administered by untrained individuals or when food and drug administration (FDA) guidelines are not adhered to. This issue may potentially be compounded by a growing reliance on social media platforms for health information.

**Objective:**

Our objective was to analyze the quality of health information in videos published on dermal filler on TikTok.

**Methods:**

We searched three hashtags, #filler (2.4 billion views), #dermalfiller (132.8 million views), and #fillersinjection (137.0 million views) and assessed the top videos returned by TikTok's algorithm that met inclusion criteria. The quality of health information was evaluated using the DISCERN instrument, a validated tool that uses a 1 to 5 scale to assess consumer health information.

**Results:**

Videos received a mean DISCERN score of 1.64 (SD 0.33), indicating significantly low quality. 7% of the videos promoted non‐FDA‐approved uses of filler. Notably, videos posted by physician assistants or physicians received the highest mean scores (1.92 and 1.72) as well as videos categorized as educational (1.99).

**Conclusion:**

Dermatologists should be aware of the high viewership of low‐quality TikTok videos on dermal filler. Dermatologists shall, therefore, understand the importance of their role in providing education to patients on this topic.

## Graphical abstract

This study analyzed the quality of TikTok's most popular dermal filler videos, revealing they are of significantly poor quality. Notably, videos posted by physician assistants or physicians, and those categorized as educational, demonstrated the highest quality. Dermatologists must acknowledge the prevalence of low‐quality TikTok content on dermal fillers and recognize their crucial role in educating patients to mitigate misinformation.

**What is already known about this topic?**

- Previous studies show that health information in TikTok videos on plastic and cosmetic surgery is of poor quality.

**What does this study add?**

- We find that the quality and reliability of patient health information in videos on dermal filler injection is also poor. Dermatologists should discourage the use of TikTok as a patient educational source.

## 1 INTRODUCTION

Dermal filler injection is currently one of the most popular minimally invasive cosmetic procedures and there has been an associated exponential growth in the market for dermal filler products.^1^ In 2020 alone, the American Society of Plastic Surgeons reported that there were over 3.4 million soft tissue filler procedures performed in the United States.^2^ The food and drug administration (FDA) has approved the use of multiple absorbable materials in adults (over the age of 21) including hyaluronic acid, calcium hydroxyapatite, and poly‐l‐lactic acid. These materials are approved for the following purposes: addressing moderate to severe facial wrinkles and skin folds such as nasolabial folds and perioral lines; augmentation of lips, cheeks, chin, and the back of the hand; correction of signs of facial fat loss in individuals with HIV; correction of contour deficiencies like wrinkles and acne scars. On the other hand, the approval for non‐absorbable fillers is limited to polymethylmethacrylate beads, specifically for addressing nasolabial folds and cheek acne scars.^3^, ^4^ Ensuring that dermal fillers are administered by trained medical professionals following FDA guidelines is crucial for maximizing safety and efficacy.

However, dermal fillers are frequently offered outside of medical settings by untrained individuals and there exists a lack of quality checks on the actual filler substance.^5^, ^6^ This trend is further compounded by the fact that cosmetic patients as well as cosmetic providers are increasingly referring to social media platforms for health information. Among these platforms, TikTok has emerged as one of the most popular, with over one billion active users.^7^ Notably, Beauty and Skincare is one of TikTok's most viewed categories, with over 33 billion views.^8^ TikTok is a user‐centered platform that allows any user to upload and share their own content. Therefore, our objective was to assess the reliability of TikTok as a source of quality health information on dermal filler through a cross‐sectional analysis of videos on dermal filler published on TikTok.

## 2 MATERIALS AND METHODS

**FIGURE 1:**
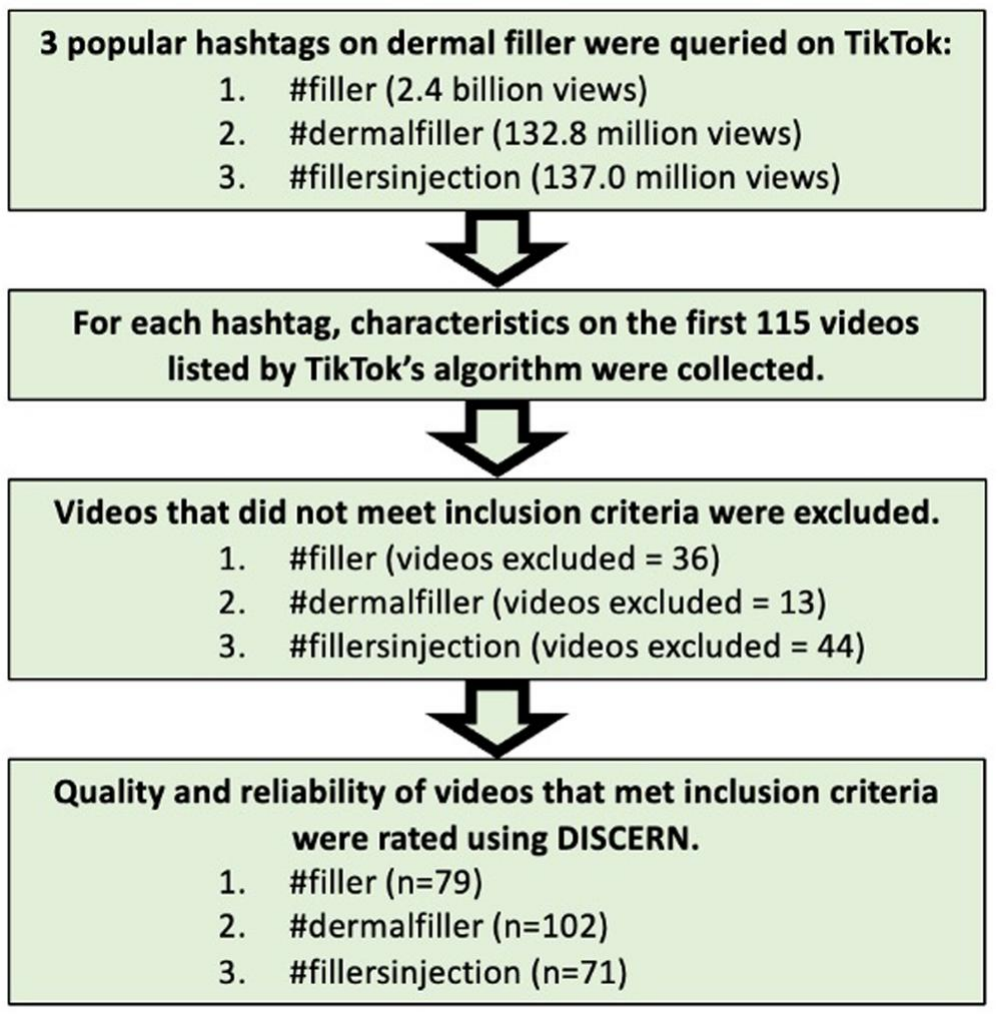
Study procedure outline.

Three relevant hashtags were searched on TikTok including #filler (2.4 billion views), #dermalfiller (132.8 million views), and #fillersinjection (137.0 million views) and for each hashtag, links for the top 115 videos returned by TikTok's search algorithm were collected between 14 December 2022 and 21 December 2022. Out of the top 115 videos per hashtag, only videos that were in English, were related to dermal filler, and were not duplicates were included. Descriptive characteristics for each video were recorded including the written caption, publication date, video duration, number of views, number of likes, number of comments, number of times favorited, number of times reposted, and content type (i.e. educational, educational/personal testimony, educational/promotional, promotional, promotional/personal testimony, comedy, comedy/personal testimony, comedy/promotional, and personal testimony). Characteristics of the uploader account were also recorded including username, account type (i.e. Physician, Physician Assistant, Nurse [includes registered nurses, nurse injectors, nurse practitioners, and nurses], Aesthetician [includes medical injectors, aesthetician experts, aesthetician practitioners, cosmetic treatment providers, cosmetic injectors, licenced aestheticians, injectors, and master injectors], Clinic [includes spas, clinics, plastic surgery practices, and dermatology clinics], Patient/General Public [includes patients and the general public], and Other [includes aesthetics training academies, pharmacists, and news outlets]), physician speciality, and presented gender. Information presented in the videos and their captions were rated by two independent reviewers using the DISCERN instrument, a validated and reliable tool for assessing consumer health information (Figure 1). The DISCERN instrument comprises of 15 questions that each represent various quality criterion and an additional 16th question that provides an overall quality rating. Each question is rated on a 1‐5 Likert scale, with one indicating no and five indicating yes.^9^ Inter‐rater reliability was tested using Pearson's correlation coefficient. Statistical analyses including one‐way ANOVAs with Tukey's multiple comparisons test and unpaired *t*‐tests were run in Prism Version 9.5.1.

## 3 RESULTS

Videos included in the analysis had a total of 212 978 631 views and 13 426 925 likes. The mean DISCERN score for the videos was 1.64 (SD 0.33), indicating they were of significantly low quality (scale 1–5). There was a high level of inter‐rater agreement, with a calculated Pearson's coefficient of 0.869 (*p* < 0.001). DISCERN criteria that had the lowest mean scores were: Does it describe what would happen if no treatment is used? (1.01), Is it clear when the information used or reported in the publication was produced? (1.02), and tied for third: Does it provide details of additional sources of support and information? and Does it provide support for shared decision‐making? (1.04).

Videos were categorized by uploader account type into seven groups: Physician, Physician Assistant, Nurse, Aesthetician, Clinic, Patient/General Public, and Other. Videos posted by Physician Assistant accounts had the highest mean DISCERN score (1.92) while videos posted by Clinic accounts had the lowest mean DISCERN score (1.53) (Table 1). A one‐way ANOVA with Tukey's multiple comparisons test among different account types (7) revealed that there were no statistically significant mean differences in the DISCERN scores between account types except for Clinic versus Physician Assistant (*p* = 0.011). When grouped together, videos posted by Physician or Physician Assistant accounts had a significantly higher mean DISCERN score than the rest of the videos posted by other account types in an unpaired *t*‐test (*p* = 0.0077). When grouped alone in an unpaired *t*‐test, videos posted by Physician Assistant accounts also had a significantly higher mean DISCERN score than the rest of the videos (*p* = 0.0056).

**TABLE 1:** ‘Mean DISCERN scores and popularity by uploader type and video category’.

	Videos	Average # of views	Average # of likes	Mean DISCERN score
Uploader type				
Physician	37 (15%)	583 079	28 815	1.72 (0.38)
Physician assistant	10 (4%)	219 324	18 413	1.92 (0.26)
Nurse	34 (13%)	628 721	56 524	1.65 (0.33)
Aesthetician	28 (11%)	848 551	36 480	1.69 (0.35)
Clinic	58 (23%)	1 239 075	60 724	1.53 (0.29)
Patient/General public	80 (32%)	888 566	70 861	1.63 (0.26)
Other	5 (2%)	224 780	8505	1.83 (0.79)
Video category				
Educational	54 (21%)	612 055	24 565	1.99 (0.32)
Educational/personal testimony	8 (3%)	363 346	25 633	1.93 (0.36)
Educational/promotional	37 (15%)	1 810 195	117 927	1.64 (0.24)
Promotional	69 (27%)	526 535	25 206	1.47 (0.21)
Promotional/personal testimony	7 (3%)	81 171	3484	1.79 (0.37)
Comedy	10 (4%)	1 700 380	158 022	1.25 (0.19)
Comedy/personal testimony	2 (1%)	45 200	2396	1.30 (0.02)
Comedy/promotional	9 (4%)	292 871	20 582	1.32 (0.16)
Personal testimony	56 (22%)	953 830	71 396	1.60 (0.23)

*Note*: DISCERN scores range from 1 (low overall quality) to 5 (high overall quality). DISCERN scores are presented as mean (standard deviation).

Videos were also grouped by content type into nine categories: Educational, Educational/Personal Testimony, Educational/Promotional, Promotional, Promotional/Personal Testimony, Comedy, Comedy/Personal Testimony, Comedy/Promotional, and Personal Testimony. A one‐way ANOVA with Tukey's multiple comparisons tests among different video categories (9) was significant (*p* < 0.0001). Videos categorized as entirely educational had the highest mean DISCERN score (1.99) while videos categorized as comedy/personal testimony had the lowest mean DISCERN score (1.30) (Table 1). When compared against all other videos using an unpaired *t*‐test, educational videos had a significantly higher mean DISCERN score (*p* < 0.0001). When comparing the prevalence of educational videos posted by Physician Assistant versus Clinic accounts, 70% of the Physician Assistant videos were categorized as entirely educational while only 5.2% of the Clinic videos were categorized as entirely educational. Conversely, only 10% of the Physician Assistant videos were categorized as entirely promotional while 55.2% of the Clinic videos were categorized as entirely promotional (Table 2).

**TABLE 2:** ‘Breakdown of uploader type by video category’.

	Educational	Educational/personal testimony	Educational/promotional	Promotional	Promotional/personal testimony	Comedy	Comedy/personal testimony	Comedy/promotional	Personal testimony
Physician	16 (43.2%)	‐	5 (13.5%)	15 (40.5%)	‐	‐	‐	1 (2.7%)	‐
Physician assistant	7 (70.0%)	‐	2 (20.0%)	1 (10.0%)	‐	‐	‐	‐	‐
Nurse	9 (26.5%)	1 (2.9%)	6 (17.6%)	14 (41.2%)	1 (2.9%)	1 (2.9%)	‐	1 (2.9%)	1 (2.9%)
Aesthetician	13 (46.4%)	‐	6 (21.4%)	2 (7.1%)	2 (7.1%)	‐	‐	3 (10.7%)	2 (7.1%)
Clinic	3 (5.2%)	‐	18 (31.0%)	32 (55.2%)	‐	‐	‐	4 (6.9%)	1 (1.7%)
Patient/General public	4 (5.0%)	6 (7.5%)	‐	4 (5.0%)	4 (5.0%)	8 (10.0%)	2 (2.5%)	‐	52 (65.0%)
Other	2 (40.0%)	1 (20.0%)	‐	1 (20.0%)	‐	1 (20.0%)	‐	‐	‐

*Note*: Percentages represent the proportion of videos of a specific content category relative to the total videos in the indicated uploader group.

There were no significant differences in mean DISCERN scores between accounts of different presented gender types. Out of the videos posted by Physician accounts, a one‐way ANOVA with Tukey's multiple comparisons test among different physician specialities (9) revealed that there were no statistically significant mean differences in the DISCERN scores between physician specialities.

The average video duration for each hashtag was 39 s (#filler), 37 s (#dermalfiller), and 41 s (#fillersinjection) and the overall average video duration was 39 s. Eighteen videos (7%) included or mentioned celebrities (6 from #filler, 6 from #dermalfiller, and 6 from #fillersinjection). Eighteen videos (7%) explicitly promoted non‐FDA‐approved methods (4 from #filler, 7 from #dermalfiller, and 7 from #fillersinjection) including injecting the nose (*n* = 6), ear lobe (*n* = 1), breast (*n* = 1), buttocks (*n* = 4), penis (*n* = 1), glabella (*n* = 2), under eyebrow (*n* = 1), temple (*n* = 1), and hips (*n* = 1) with dermal filler. Videos featuring non‐FDA‐approved methods had a total of 5 532 291 views and 559 635 likes.

## 4 LIMITATIONS

TikTok's algorithm for selecting a hashtag's top videos is dynamic, meaning that these top videos are constantly changing. The study time frame is reflected in the methods however we recognize that results may variable depending on when a similar analysis may be performed. Additionally, data collected on uploader account type relies solely on what the accounts self‐report, introducing possibility for inaccuracy. We also assumed that the uploader account was the primary producer and publisher of the videos. However, in this study it was noted that patient/general public uploaded videos (32%) were represented more than videos by other uploader group types.

## 5 DISCUSSION/CONCLUSION

TikTok is becoming an increasingly popular source for health information as social media usage continues to rise worldwide.^10^ In the context of TikTok's popularity and the prevalence of dermal filler content on TikTok, this is the first study to investigate the quality of health information in TikTok videos on dermal filler. Our analysis found that videos categorized as educational received the highest quality mean score (1.99 (SD 0.32)). This reflects the results of a previous study that looked at the quality of plastic and cosmetic surgery videos on TikTok and found that videos categorized as educational scored the highest.^11^ Notably, our study found that only 21% of the most popular dermal filler TikTok videos were categorized as educational.

Upon analyzing the TikTok videos based on uploader type, we found that videos posted by Physician Assistant or Physician accounts received the highest and third‐highest quality scores, respectively. In contrast, videos posted by Clinic accounts received the lowest quality scores. This may be due to over half of the videos posted by Clinic accounts being categorized as entirely promotional (55.2%), whereas only a small fraction (5.2%) of their videos were classified as entirely educational. In comparison, the majority of videos posted by Physician Assistant accounts were categorized as entirely educational (70%), whereas only 10% were categorized as entirely promotional. Promotional videos are often designed to attract viewership and encourage the use of dermal filler and are therefore a poor source for unbiased information. In contrast, educational videos are more likely to provide higher quality health information as they do not need to promote a product, allowing for greater objectivity.

When investigating the prevalence of videos with potentially unsafe or misleading information on dermal filler, we found that 18 videos (7%) promoted non‐FDA‐approved uses of dermal filler and garnered a total of a total of 5 532 291 views and 559 635 likes. Among these, the application of filler for nose augmentation (*n* = 6) and buttocks enhancement (*n* = 4) emerged as the most frequently featured applications in these videos. Notably, only three out of the 18 videos were posted by Physician accounts. The rest were posted by either Aesthetician, Clinic, or Patient/General Public accounts. Considering this along with the findings that educational videos and videos posted by Physician Assistant and Physician accounts had the highest quality ratings, we recommend that highly trained medical professionals create and share more TikTok videos with educational intent.

Overall, the quality of the analyzed videos was low, as measured by the DISCERN scale. DISCERN criteria that received the lowest scores were describing what happens if there is no treatment, indicating the date when the information was produced, providing additional sources of support and information, and providing support for shared decision making. While it may not be meaningful to discuss the outcome of not receiving dermal filler treatment due to its elective and temporary nature, the other criteria are important to address. One challenge to including the other criteria may be the short length of TikTok videos. However, TikTok allows users to add written captions to their videos which can be used to include citations, dates, and sources of additional information without increasing the video duration or disrupting the viewer engagement. Regarding the last DISCERN criteria of providing support for shared decision making, it's crucial for the public to understand the importance of engaging in shared decision making with licenced medical professionals. Dermal filler injection holds serious risks and possible complications, such as accidental injection into a blood vessel which can result in skin necrosis, stroke, or blindness. Furthermore, removal of fillers requires additional procedures with their own risks and may not always be possible.^4^, ^12^ Therefore, to improve the quality of dermal filler TikTok videos, we recommend that the video component incorporates a message promoting shared decision‐making in addition to a utilization of captions for supplementary information and references.

TikTok provides an excellent opportunity for medical professionals to reach patients and provide valuable educational health information. However, currently, the majority of TikTok videos suggested by TikTok's algorithm on dermal fillers are posted by unlicensed individuals, lack quality, and sometimes promote non‐FDA‐approved methods. This underscores the need for more qualified and highly trained medical professionals to share high‐quality educational content that includes proper citation information, additional sources of information, and support for shared decision making.

## CONFLICT OF INTEREST STATEMENT

None to declare.

## AUTHOR CONTRIBUTIONS

**Heloise de Baun**: Conceptualization (lead); Data curation (lead); formal analysis (lead); investigation (lead); methodology (lead); project administration (lead); resources (equal); software (equal); supervision (equal); validation (lead); visualization (lead); writing – original draft (lead); writing – review & editing (lead). **Patricia Cerri‐Droz**: Conceptualization (equal); data curation (equal); formal analysis (equal); investigation (equal); methodology (equal); project administration (equal); resources (equal); supervision (equal); validation (equal); visualization (equal); writing – original draft (equal); writing – review & editing (equal). **Samavia Khan**: Conceptualization (supporting); data curation (equal); formal analysis (equal); funding acquisition (equal); investigation (equal); methodology (equal); project administration (equal); resources (equal); supervision (equal); validation (equal); visualization (equal); writing – original draft (equal); writing – review & editing (equal). **David Alper**: Conceptualization (supporting); data curation (supporting); formal analysis (equal); investigation (equal); methodology (equal); project administration (equal); resources (equal); validation (equal); visualization (equal); writing – original draft (supporting); writing – review & editing (supporting). **Barbar Rao**: Conceptualization (equal); data curation (equal); formal analysis (equal); investigation (equal); methodology (equal); project administration (equal); resources (equal); supervision (equal); validation (equal); visualization (equal); writing – review & editing (equal).

## ETHICS STATEMENT

Not applicable.

## Data Availability

The data used to support the findings of this study are publicly available on the social media application TikTok and associated website.
